# We bid farewell to Prof. Dr. med. Wolf Langewitz (27.10.1951–28.12.2024)

**DOI:** 10.3205/zma001738

**Published:** 2025-02-17

**Authors:** Claudia Kiessling, Cadja Bachmann, Alexander Benz, Götz Fabry, Annette Fröhmel, Anja Härtl, Linn Hempel, Henrike Hölzer, Rolf Kienle, Heiderose Ortwein, Bärbel Otto, Tim Peters, Swetlana Philipp, Susanne Pruskil, Katrin Rockenbauch, Kai P. Schnabel, Monika Sennekamp, GMA Committee for Communication and Social Competencies

**Affiliations:** 1 Witten/Herdecke University,Faculty of Health, Chair for the Education of Personal and Interpersonal Competencies in Health Care, Witten, Germany; 2 University Medical Centre Rostock, Rostock, Germany; 3 Ludwig-Maximilians-Universität, Faculty of Medicine, Institute of Medical Psychology, Munich, Germany; 4 Albert-Ludwig-Universität, Faculty of Medicine, Department of Medical Psychology, Freiburg i. Brsg., Germany; 5 Academy of Public Health Services, Düsseldorf, Germany; 6 University Hospital Augsburg, Department of Hygiene and Environmental Medicine, Augsburg, Germany; 7 University of Halle-Wittenberg, Medical Faculty, Dorothea Erxleben Learning Centre, Halle (Saale), Germany; 8 Berlin, Germany; 9 Charité – Universitätsmedizin Berlin, Prodekanat für Studium und Lehre, Berlin, Germany; 10 Center for Pain Medicine Ostseestraße, Berlin, Germany; 11 LMU Munich, University Hospital, Institute of Laboratory Medicine, Munich, Germany; 12 Bielefeld University, Medical School OWL, Bielefed, Germany; 13 University Hospital Jena, Institut für Psychosoziale Medizin, Psychotherapie und Psychoonkologie, Jena, Germany; 14 Local Health Authority – Altona, Hamburg, Germany; 15 Geschäftsstelle Hochschuldidaktik Sachsen (HDS), Leipzig, Deutschland; 16 University of Bern, Institute for Medical Education, Bern, Switzerland; 17 Goethe University Frankfurt, Institute of General Practice, Frankfurt/Main, Germany

## Prof. Dr. med. Wolf Langewitz (27.10.1951–28.12.2004)

It is with sadness and gratitude that we, the members of the “Communicative and Social Competencies Committee” (KusK) of the DACH Association for Medical Education (GMA), bid farewell to Prof. Dr. Wolf Langewitz.

## The teacher and mentor

For many of us, Wolf was a highly valued colleague, a teacher and mentor, a companion and trailblazer, a source of inspiration and a friend. In 2005, he co-founded the KusK Committee, a committee that continues to lead the way in teaching, research and collegial collaboration on the subject of communication. The first KusK workshop took place in 2006 in Basel, Wolf's adopted home. With the support of the Carl Gustav Carus Foundation, which Wolf chaired for many years, two further KusK workshops were realized. These laid the foundation for the Committee’s successful work to this day. Wolf enriched our workshops and the committee's work, for example with a lecture on the Basel communication curriculum “SoKo” (2008 in Undeloh) and a workshop on working with critical incidents (2017 in Bad Schandau). He accompanied us professionally and personally – directly through his advice or his impressive workshops and indirectly through his numerous publications on the topic of clinical communication on our (sometimes rocky) path to establishing longitudinal communication curricula at many medical faculties in Germany and Switzerland. In doing so, he not only focused on professional aspects, but also always had the students and us teachers in mind. Wolf's appreciative and constructive feedback was orientated towards individual needs and the resources on site. 

## The trailblazer of skills- and evidence-based teaching of communication skills

Wolf pursued a skills-based approach to teaching communication skills without losing sight of the importance of person- and relationship-orientated medicine. For Wolf, clinical communication was something to be learned and was an indispensable part of medical professionalism. Communication techniques such as “WRRS” (Wait, Repeat, Reflect, Summarize, WWSZ in German), the “book metaphor” and working with critical incidents, which are now part of the basic teaching repertoire at many sites, are largely credited to Wolf. Mindful listening, “just shut up”, the “Langewitz breathing break” were key recommendations for students and doctors alike to enable patients to articulate their concerns and express their worries and emotions.

His teaching videos, the DocCom.Deutsch modules – designed and developed in collaboration with Christoph Daetwyler, the Institute for Medical Education (Bern) and many other communication instructors – and his publications, e.g. on the Basel Ward Round Standard, accompany us in our work and are an essential basis for evidence-based teaching and development of communication skills. He contributed his extensive expertise and set standards not only in teaching, but also in assessment of communicative competencies. Wolf considered it essential to substantiate teaching and assessment scientifically, underlining his importance as a scholar and researcher in this field.

The qualification of doctors and teachers was also an important concern for him. His train-the-trainer courses were impressive, multi-faceted and lasting. Wolf visited many of us in person and supported the conceptualization and further development of the communication curricula through his practical and interactive workshops. It was important to him to start with the basics without overtaxing the students, i.e. not trying to immediately conquer Eiger’s North Face, but to take a hike in the Harz Mountains first, as he loved to put it.

## The thinker – “Verschmitztes” and neo-phenomenology

We were not always ready to follow his intellectual paths. His enthusiasm for philosophical excursions, especially into neo-phenomenology in recent years, overwhelmed some of us at times. Terms such as “composure” (Fassung), “constellation” (Konstellation) or “lived body” (Leib) often raised eyebrows and caused questions. He was forgiving, the similarities were always far greater than the differences.

## The humorous, curious and generous person

Wolf was not only a competent and inspiring teacher and mentor. We will also miss the humorous, curious and generous person Wolf was. He treated everyone who asked him for advice with great appreciation. No question was too small for him, no colleague too new to the field. With a twinkle in his eye and a sense of humor, he commented on the oddities of academia and encouraged us to overcome the many hurdles that were sometimes put in our way in the scientific or clinical world. 

Above all, Wolf was generous. We were all allowed to use his material, videos and course instructions, including unpublished material. He always shared his knowledge and experience to help and support others in their teaching and career planning. In a world in which hardly anything counts apart from funding and publications, and in which content is often closely safeguarded, this openness and generosity was extraordinary and remarkable. We are inspired by and very grateful for this.

## The visionary – for a person- and relationship-centered medicine

Wolf’s important commitment to clinical communication has improved medical education and patient care. His vision, his perseverance and his wealth of ideas will continue to encourage us to stand up for a person- and relationship-centered medicine and teaching in the future. His attitude to always keep this overarching goal in mind was both effective and impressive, and left its mark on the collegial development of the committee. The number of patients who as a result have been, are and will be able to better communicate their concerns to their doctors is almost incalculable and certainly substantial.

## In gratitude

Our deepest sympathies goes to his family and all those who knew and will miss him. Wolf is no longer where he was, but he is wherever we teach communication and pass on his legacy to the next generations.

We have learned so much from you.

Claudia Kiessling, Cadja Bachmann, Alexander Benz, Götz Fabry, Annette Fröhmel, Anja Härtl, Linn Hempel, Henrike Hölzer, Rolf Kienle, Heiderose Ortwein, Bärbel Otto, Tim Peters, Swetlana Philipp, Susanne Pruskil, Katrin Rockenbauch, Kai Schnabel, Monika Sennekamp, on behalf of the KusK committee of GMA.

## Authors’ ORCIDs

- Claudia Kiessling: [0000-0003-4104-4854]
- Cadja Bachmann: [0000-0001-8092-1309]
- Alexander Benz: [0000-0001-6622-3715]
- Götz Fabry: [0000-0002-5393-606X]
- Linn Hempel: [0009-0009-5421-2029]
- Rolf Kienle: [0000-0003-2253-8832]
- Susanne Pruskil: [0000-0002-9264-9784]
- Katrin Rockenbauch: [0009-0009-5934-6413]
- Kai P. Schnabel: [0000-0002-6977-2717]

## Competing interests

The authors declare that they have no competing interests. 

